# Characterisation and utilisation of nano-reduced starch from underutilised cereals for delivery of folic acid through human GI tract

**DOI:** 10.1038/s41598-021-81623-8

**Published:** 2021-03-01

**Authors:** Faiza Jhan, Asir Gani, Nairah Noor, Zanoor ul Ashraf, Adil Gani, Asima Shah

**Affiliations:** 1 Department of Food Science and Technology, University of Kashmir, Srinagar, 190006 India; 2 Department of Food Science, Rutgers University, 65 Dudley Road, New Brunswick, NJ 08901 USA

**Keywords:** Nanoparticles, Polysaccharides

## Abstract

Ball milling offers green approach for size reduction of starch granules to nano scale size. In this research work, the starch from two underutilised cereal varieties viz. foxtail starch (FS) and sorghum starch (SS) were milled to achieve the desired nanometric range with mean particle diameter of 467.98 and 271.12 nm for nano foxtail (FSN) and nano sorghum starch (SSN), which were highly stable as revealed by zeta potential analysis. Functional attributes like solubility, swelling index, apparent amylose content, emulsifying and pasting properties were evaluated. Scanning electron microscopy (SEM) clearly revealed damaged starch granules produed by the process of milling. X-ray diffraction (XRD) displayed decrease in crystallinity upon milling to 16.08% (SSN) and 18.56% (FSN) and disappearance of some peaks. Attenuated total reflectance-fourier transform infrared spectroscopy (ATR-FTIR) also revealed reduced crystallinity as confirmed by the decreased absorbance ratio of 1047/1022 cm^−1^ in nano starch particles. Rheological analysis displayed shear thinning behaviour of nano starch samples as evaluated using Herschel-bulkely model and Power law. The nano starch samples exhibited comparatively low thermal gelatinisation temperatures as compared to native counter particles. Moreover, the nano-encapsulated starch samples offered more resistance to in-vitro digestion and showed control release of folic acid at target sites.

## Introduction

Starch is a carbohydrate biopolymer, non-toxic in nature, highly biodegradable and an inexpensive polysaccharide abundantly present in plants, cereals, crops and fruits has grabbed much attention in recent years due to its wide applications. Structurally, it is semi-crystalline composed of an amorphous (amylose) and non-amorphous (amylopectin) region whose proportions depend on factors like the botanical source, degree of plant maturity and cultivar differences of the same species^1,2^. It has extensive applications as raw material in different food, pharmaceutical and textile industries due to its properties like biocompatibility, storage stability, less toxicity and excellent drug delivery vehicle^3^. This constantly mounting usage of starch is leading a way to modify this essential polymer in order to meet specific industrial requirements^4^. One such modification is nano-reduction of this macromolecule, which is trending now-a-days due to its customised small size, improved functional attributes, greater mechanical strength, better bioactive retention, improved and controlled release behaviour of encapsulated functional ingredients leading to significant improvement in various applications of this polysaccharide^5^. However, the method of size reduction of this polymer into nanometric range plays a significant role due its impact on the polymer functionality, yield and time consumption. In order to minimise changes in the basic characteristics of starch polymer simple physical nano-reduction method i.e., ball milling provide benefits over other reduction methods which include simple operating process, less contamination chances, high size reducing frequency and yield^6^. Moreover, nanotechnology is being used in various fields especially in drug and food industries in order to enhance the stability of various unstable nutrients or bioactives. Folic acid (*N*-[4-[[(2-amino-1,4-dihydro-4-oxo-6-pteridinyl) methyl]amino]benzoyl]-l-glutamic acid), a vital water soluble natural vitamin present in various foods like green vegetables and fortified foods^7^ promotes the red blood cells formation and is also regarded as an anti-anaemia factor. Furthermore, it averts neural tube defects, Alzheimer’s disease, cardiovascular diseases, pregnancy-related complications etc^8,9^. In spite of these advantageous properties there are certain limitations associated with this bioactive like under acidic environment its easy degradation, hence low bioavailability^8^. Therefore, to increase the bioavailability and bio accessibility of folic acid it needs to be protected under harsh conditions. Nano-encapsulation could be an interesting approach to safeguard this essential nutrient from degradation leading to its high surface-to-volume ratio for improved intracellular penetration, higher bioavailability as well as its gradual release at target sites^9–11^. The daily requirement of folic acid in a normal adult is about 200 μg daily which needs a high dose to meet such prerequisite. The current approach can significantly decrease the dose requirement of this essential vitamin to meet the daily body demand. With these advantages of nano biopolymers like simple organisation, nano-molecular complexes formation and desirable functionality they are receiving prodigious attention compared to the complex structured polysaccharides. Therefore, the current work targets to study changes in functional properties, structural attributes, thermal properties, and rheological characteristics of starch extracted from two minor cereal varieties i.e., sorghum and foxtail millet when reduced to nano range. The study also aims to compare performance of starch from both varieties as wall materials for folic acid and its in vitro release behaviour in human GI tract conditions.

## Results and discussion

### Particle size and zeta potential

The mean particle size and zeta potential of native and nano starch granules is shown in Table 1. The average particle size of SS and FS was found to be 2076.73 nm and 1784.89 nm which when subjected to physical agitation by ball milling resulted in the fragmentation of macro starch granules with reduced average size of 467.98 nm and 271.12 nm for FSN and SSN. The average particle size of native starch granules was found comparatively lower in FS while as in case of nano-starch samples it was found lowest in SSN. The factors that play key role in fragmentation and size reduction of particles include the difference in the origin of cereals, composition of starch, amylopectin branching etc^12^. Moreover, millet starches exhibit A-type starch pattern^13^, which is easily susceptible to breakage due to the presence of unbranched amylopectin portion when subjected to shear forces during milling^14^. Changes in the zeta potential values was revealed by the DLS. The zeta potential values were found to be negative in all the samples under investigation. The surface charge of FS and SS was found to be − 44.64 mV and − 21 mV which increased to − 24.27 mV and − 12.16 mV for FSN and SSN, indicating a shift towards greater stability and less possibility of aggregation^15^. Overall, SS revealed the highest stability both in the macro and nano cases. The increased exposure of hydroxyl groups (–OH) on starch nanoparticles when subjected to ball milling process might have contributed to the increase in zeta potential^16^.

**Table 1:** Particle size, zeta potential, molecular order obtained by ATR-FTIR and relative crystallinity quantified by XRD of native and nano starch samples.

Parameters	FS	FSN	SS	SSN
Particle size (nm)	1784.89^c^	467.98^b^	2076.7^d^	271.12^a^
Zeta potential (mV)	− 44.64^d^	− 24.27^c^	− 21^b^	− 12.16^a^
1047/1022^A^ (cm^−1^)	0.69	0.41	0.89	0.43
Relative crystallinity (%)	33.48%	18.56%	30.23%	16.08%

Values expressed are mean ± standard deviation. Means in the row with different superscript (a, b, c & d) are significantly different at P ≥ 0.05. FS, FSN, SS and SSN represent native foxtail millet starch, nano foxtail millet starch, native sorghum starch and nano sorghum starch, respectively.

^A^ The ratio of absorbances at wavenumbers 1047/1022.

### Surface morphology of starch sample

**Figure 1:**
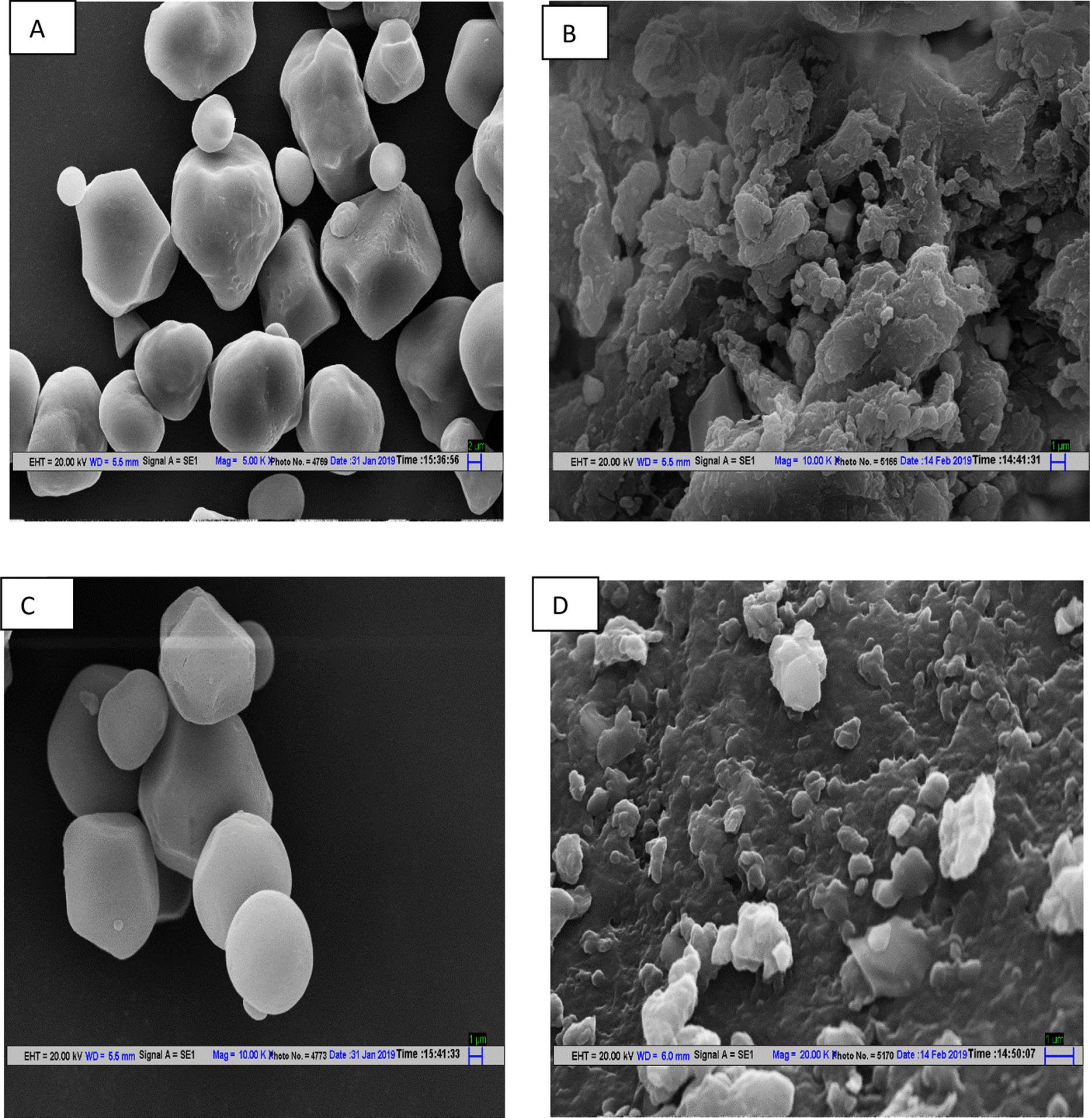
**(a–d)** Scanning electron micrographs of native foxtail starch **(a)** nano-reduced foxtail starch **(b)** native sorghum starch **(c)** and nano sorghum starch **(d)**.

The granular surface topographic images of native and ball milled starch samples are displayed in Fig. 1(a–d). The granules of FS were found to be polygonal with few very small round shaped smooth granules at surface however SS was mostly polyhedral in shape with some smooth spherical shaped granules also present. Difference in the shape and size of native millet starches might be due to the difference in their botanical origin, plant physiology or the biochemical difference of the amyloplast or chloroplast ^17^. The ball milling treatment bought significant changes in the granular structure of starch breaking it into very small fragments and disarraying the smooth and flat surfaces into irregular and rough surfaces. As can be seen, the fragmentation of SSN is more pronounced when compared to FSN which was also revealed earlier by the average particle size analysis. The small granules have grooves and fissures on the surface without any clear defined granular structure in both the cases. The milling process affected the granules inducing the irregularities to the surfaces and converting the granules into nano-range, thereby affecting the physico-chemical and functional properties of the starch particles. Furthermore, it can be clearly seen that the starch granules exposed to process of ball-milling tend to clump together forming cluster which might be attributed to the agglomeration of damaged granules due to the rupturing of hydrogen bonds present in the granules which possibly link with each other forming the lumps^18^. The results are in good agreement with the data obtained in DLS. Similar results were observed in maize starch by Liu et al^19^ after ball milling treatment.

### Crystallographs of starch samples

**Figure 2:**
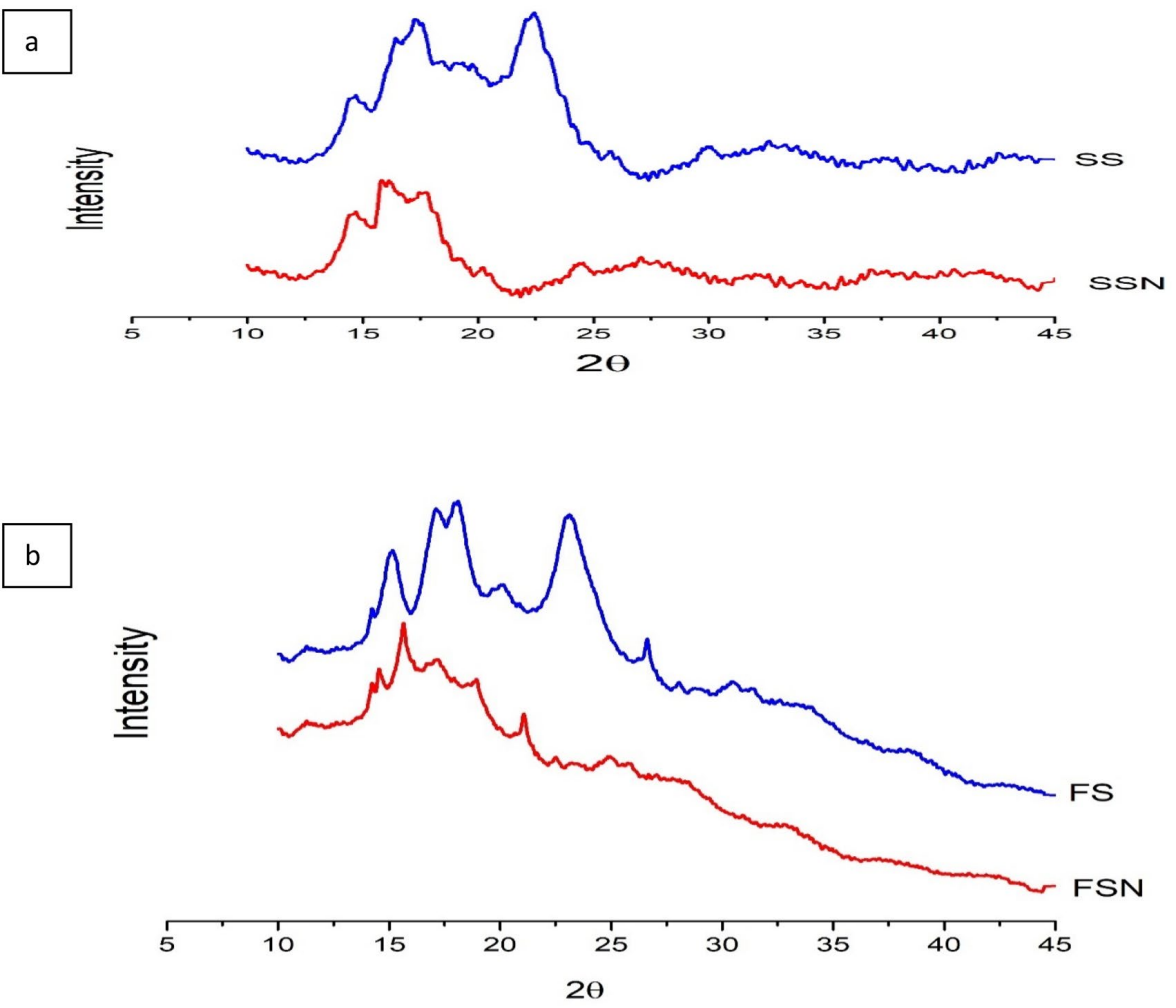
**(a,b)** Crystallinity graphs of native and nano starch samples. SS, SSN, FS & FSN represents native sorghum starch, nano sorghum starch, native foxtail starch & nano foxtail starch respectively.

The basic polymorphic pattern of native and nano starch of both varieties is shown in Fig. 2(a,b) and the change in their relative crystallinity is given in Table 1. Starch diffraction pattern mainly include A, B and C types. The crystallographic pattern of SS revealed one strong diffraction peak at around 22.2°, two strong unresolved peaks at around 19.22° and 17.3°, and one another slight peak at 14.7° Bragg angle (2θ) depicting a typical A-type pattern of diffraction^20^ (Fig. 2a). The similar A-type X-ray diffraction peak pattern was also revealed in FS with a prominent peak at 26.61°, two unresolved peaks at 17.17° and 18°, another peak at 15.14° and a slight peak at around 23.11° (Fig. 2b). The SSN displayed decreased intensity and disappearance of some peaks which include a total loss of diffraction peak around 22.2° indicating the total structure interruption. The similar results were also found in FS upon milling for 1 h. Moreover, the relative crystallinity (RC) decreased from 30.23% (SS) and 33.48% (FS) to 16.08% (SSN) and 18.56% (FSN) upon reduction in particle size of starch granules. The underlying reason for intensity reduction and loss of some diffraction peaks might be attributed to the disruption of ordered 3-D structure of amylopectin and formation of more amorphous character of starch particles due to the particle size reduction by milling process ^21^. He et al. ^22^ also reported the loss of the diffraction peaks and decreased crystallinity in maize starch when subjected to steel ball milling.

### FTIR analysis

**Figure. 3:**
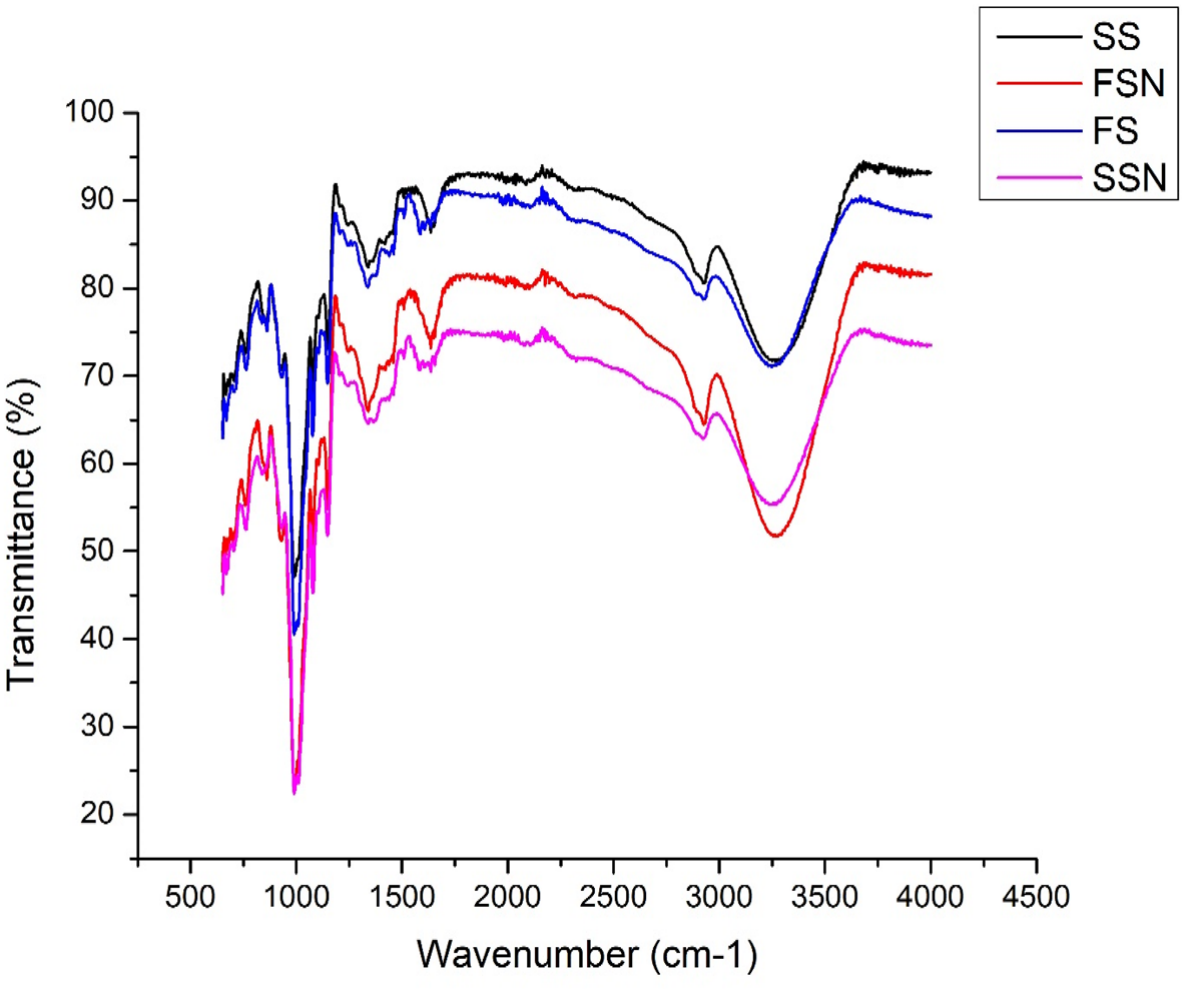
ATR-FTIR spectra of native and nano starch samples. FS, FSN, SS and SSN represent native foxtail millet starch, nano foxtail millet starch, native sorghum starch and nano sorghum starch, respectively.

The results of infrared spectroscopy revealed the structural changes in starch granules produced by the physical agitation using ball milling method (Fig. 3). Prominent bands at around 3400–3200 cm^−1^ and 2923–2925 cm^−1^ attribute to the stretching vibration of hydroxyl groups and C–H groups respectively. The absorption band around 1645 cm^−1^ originated due to the bound water present in starch granules. The peak around 990 cm^−1^ corresponds to the characteristic α-1, 4 glycosidic linkages and C–O–C stretch^23^. No real change in the functional groups of starch samples took place upon size reduction. However, the absorption band intensity around 990 cm^−1^ decreased considerably both in SSN and FSN which might be associated with the structural disruption of inner molecular order upon milling process making it easily accessible to water. Peaks around 1047 cm^−1^ and 1022 cm^−1^ determine the crystalline and amorphous structure of the starch samples. Furthermore, the ratio of absorbances between 1047/1022 cm^−1^ determine the increase or decrease in crystallinity of granular microstructure of starch^24^ (Table 1). The decreased absorbance ratio was found in the nano-reduced starch samples compared to native forms which is an indicative of crystallinity reduction after the irreversible disruption of double helices packing of the starch molecules due to ball milling treatment leading to the generation of disordered amorphous starch granules^25^. These results are consistent with the findings of XRD. Hedayatia et al.^26^ also observed a decrease in the ratio of absorbances at 1047/1022 cm^−1^ for tapioca native and nano starch particles.

### Thermographs of starch samples

**Figure 4:**
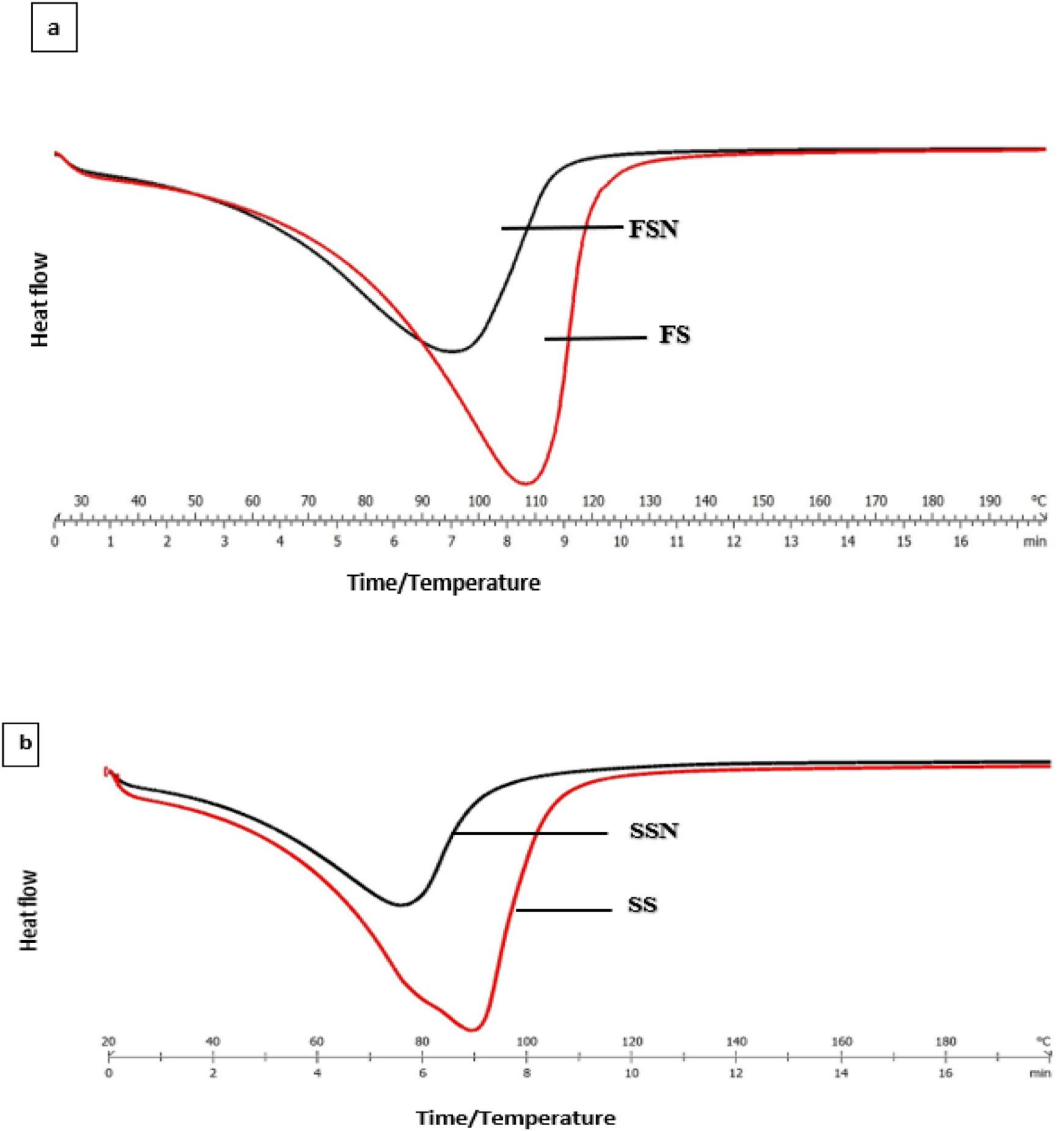
**(a,b)** Thermographs of native and nano- reduced starch samples. FS, FSN, SS and SSN represent native foxtail millet starch, nano foxtail millet starch, native sorghum starch and nano sorghum starch, respectively.

The thermographs of native and nano-reduced starch samples are depicted in Fig. 4(a,b). As revealed by the thermal curves, the phase transition temperatures of ball-milled starch samples was significantly lower than native starch samples depicting that gelatinisation of nano-reduced starch is comparatively easier. The onset gelatinisation temperature (T_o_) of SS and FS was found to be 59.07 °C and 76.13 °C which showed a shift to some lower degrees of temperature viz 47.61 °C and 58 °C when nano-sized. Similar trend was followed by the peak gelatinisation temperature (T_P_) which showed a shift from 88.8 °C (SS) to 75.37 °C (SSN) and 103.51°C (FS) to 92.47°C (FSN). The conclusion temperature (T_c_) for SS and FS was detected at 100.65 °C and 118.90 °C which also decreased to 89.34 °C and 112.34 °C for SSN and FSN, as revealed from the figure. Moreover, the endothermic enthalpy which is an indicative of crystallinity and presence of double helical structures reduced for nano-starch samples when compared to native starch samples. The gelatinisation enthalpy value reduced from 16.83 J/g (native) to 11.31 J/g (nano) for sorghum starch and 15.83 J/g (native) to 112.76 J/g (nano) for foxtail starch. The possible reason for decrease in enthalpy on nano sizing might be the fact that native starch samples are semi-crystalline in nature and have double helical structure due to which it needs higher temperature for gelatinisation. In contrast, decreased ordered structure in nano-starch and conversion to single helical structure caused by the mechanical agitation during ball milling process leads to the decrease in gelatinisation properties^27^. Similar findings of reduction in gelatinisation temperatures has also been revealed by Ding et al.^28^ for RS4 nanoparticles.

### Apparent amylose content

The amylose content present in native and nano-reduced starch samples is presented in Table 2. The measured apparent amylose content in FS and SS was found to be 30.58% and 33.54%. As per the reports, millet starches contain amylose content in the range of 31–33%. Liu et al^29^ reported 28.7% of amylose content in native sorghum starch. The amylose content difference depends on the factors like botanical origin, granule morphology of the starch and starch extraction methods^30^. After milling process, the apparent amylose content of the nano-starch particles increased significantly (p > 0.5) to 42.53% and 45.05% for FSN and SSN. The physical shear forces during milling easily destroys the branched amylopectin converting it into the smaller linear amylose units^31^. Moreover, the increase in amylose content was found higher for SSN which might also be ascribed to its greater destruction and lowest average mean particle size as already revealed by the SEM images and DLS data. The increase in amylose content upon nano-reduction can directly affect the functionality of the starch which is considered beneficial for the diabetic patients, since this kind of starch shows low glycaemic index^32^. The increase in apparent amylose content of resistant starch upon nano-reduction was also reported by Ding et al.^28^.

**Table 2:** Amylose content and color values of native and nano starch samples.

Parameters	FS	FSN	SS	SSN
Amylose content (%)	30.58 ± 0.59^a^	42.53 ± 0.50^c^	33.54 ± 0.58^b^	45.05 ± 0.25^d^
**Color**				
L*	85.14 ± 0.41^a^	90.98 ± 0.48^b^	90.23 ± 0.93^b^	94.26 ± 0.53^c^
a*	− 0.54 ± 0.36^c^	− 0.70 ± 0.13^d^	− 0.14 ± 0.12^a^	− 0.28 ± 0.10^b^
b*	11.30 ± 2.06^c^	11.23 ± 0.39^c^	9.77 ± 0.99^a^	10.72 ± 0.84^b^

Values expressed are mean ± standard deviation. Means in the row with different superscript (a, b, c & d) are significantly different at P ≥ 0.05. FS, FSN, SS and SSN represent native foxtail millet starch, nano foxtail millet starch, native sorghum starch and nano sorghum starch, respectively. ‘L*’ = lightness, ‘a*’ = redness to greenness and ‘b*’ = yellowness to blueness.

### Color

Color is considered an important parameter in food industry with high lightness values usually desirable in terms of consumer acceptability. The calorimetric values of native and nano-reduced starch granules are presented in Table 2. A significant (P < 0.05) difference among hunter color parameters viz L* (lightness), b*(redness) & a* (greenness) were noted. The samples SS and FS revealed high L* values with low chroma values i.e., a* and b* specifying that starch is whiter in color. Dey et al.^33^ observed much lower lightness value (76.20) for native foxtail starch. Moreover, a significant increase in L* value from 85.14 (FS) and 90.23 (SS) to 90.98 (FSN) and 94.26 (SSN) was detected with substantial reduction of b* value in nano starch samples. This increase in whiteness may be explained on the basis of increased surface area upon size reduction resulting in the higher refractive index values in milled starch particles. Similar increase in lightness values was observed in acid modified pearl starch by Balasubramanian et al.^34^.

## Swelling and solubility index

The swelling power and solubility index of native and nano-reduced starch samples are presented in Table 3. Apparently, the swelling index evaluated at different temperatures ranging from 60 to 90 °C showed an increasing trend in the range of 6.25–9.64%, 7.42–10.90%, 7.47–10.68% and 8.49- 12.04% for FS, FSN, SS and SSN, respectively. As temperature rises, the binding forces between starch granules weakens thus facilitating good swelling ability. The swelling index for SS was found significantly (p > 0.5) higher at all temperature ranges when compared to FS. However, the nano-counter parts depicted comparatively higher swelling index than native samples in both the cases. The swelling power directly depends on the structure, molecular weight, amylose content and shape of the starch granules^32^. Hence, the reason for the increase in swelling power on size reduction might be the shear forces during milling process which disrupts the crystalline part converting it into an amorphous structure with less amylose-amylopectin, amylose-amylose and amylopectin-amylopectin associations so that water easily gets entrapped. Similarly, the results revealed highest solubility index for native starch samples at 90 °C with SS showing high solubility (9.08%) than FS (8.04%). This corresponds to the high amylose content in sorghum starch (already discussed), since solubility of starch mainly depends upon amylose leaching^35^. The nano- reduced starch samples showed increased solubility at all temperatures compared to native starch samples. The reason attributed to this might be that upon ball-milling both intra and inter hydrogen molecular bonds weaken and more hydroxyl groups gets exposed leading to more interaction with water molecules resulting in improved solubility. However, the native samples have strong internal structure resisting the leaching of amylose and are therefore, comparatively less soluble. Similar increase in swelling and solubility upon nano-reduction have also been earlier reported for waxy rice starch upon nano-reduction using ball milling (BM) and octenyl succinic anhydride (OSA) modification^36^.

**Table 3:** Swelling, solubility and emulsifying properties of native and nano starch samples.

Parameters	FS	FSN	SS	SSN
**Swelling power (%)**				
60 °C	6.25 ± 0.38^a^	7.42 ± 0.52^b^	7.47 ± 0.41^b^	8.49 ± 0.44^c^
70 °C	7.95 ± 0.07^a^	8.86 ± 0.12^b^	8.62 ± 0.53^b^	9.59 ± 0.45^c^
80 °C	8.76 ± 0.27^a^	9.73 ± 0.36^b^	9.93 ± 0.06^b^	10.80 ± 0.23^c^
90 °C	9.64 ± 0.53^a^	10.90 ± 0.12^b^	10.68 ± 0.21^b^	12.04 ± 0.27^c^
**Solubility (%)**				
60 °C	2.09 ± 0^a^	4.13 ± 0.02^c^	3.04 ± 0.01^b^	7.19 ± 0.01^d^
70 °C	3.03 ± 0^a^	5.19 ± 0.019^c^	4.06 ± 0.01^b^	8.29 ± 0.01^d^
80 °C	5.03 ± 0^a^	6.26 ± 0.01^b^	6.07 ± 0^b^	9.36 ± 0.03^c^
90 °C	8.04 ± 0.01^a^	11.29 ± 0.01^c^	9.08 ± 0.01^b^	13.50 ± 0.02^d^
Emulsifying capacity (%)	15.42 ± 0.30^b^	18.11 ± 0.20^c^	14.85 ± 0.27^a^	19.85 ± 0.19^d^
Emulsifying stability (%)	12.69 ± 0.29^b^	14.71 ± 0.38^c^	11.89 ± 0.23^a^	14.26 ± 0.63^c^

FS, FSN, SS and SSN represent native foxtail millet starch, nano foxtail millet starch, native sorghum starch and nano sorghum starch, respectively. Values expressed are mean ± standard deviation. Means in the row with different superscript (a, b, c & d) are significantly different at P ≥ 0.05.

### Emulsifying ability and stability

To understand the effect of particle size on the emulsion forming abilities, the native and nano-reduced starch samples were analysed and the values obtained are depicted in Table 3. The emulsifying capacity and stability values for FS and SS were found to be 15.42, 14.85 and 12.69, 14.26, respectively. A gradual increase in emulsifying capacity and stability upon size reduction is seen signifying its better ability to form oil–water interface. The reduced particle size was also positively correlated with improved emulsion forming property by Falade^37^ where he found bitter yam starch with lowest particle size showed higher emulsion forming property compared to other large sized starch particles. Moreover, among all the samples analysed the emulsifying property of SSN was found to be significantly higher which is consistent with the results obtained by DLS (explained earlier) showing its lowest mean particle size diameters. Thus, ball milling can significantly improve emulsion forming ability of starches.

### Pasting properties

The pasting properties of native starch and changes upon nano-reduction was evaluated using RVA and values are presented in the Table 4. The significant reduction in the peak, trough, breakdown, final and setback viscosities were found and increase in pasting temperature was observed in starch samples upon particle size reduction (Table 1). Peak viscosity which is an indicative of rapid starch granule swelling and amylose leaching was found lowest for SSN followed by FSN. Although the disordered molecular structure of starch granules enhance the swelling ability of the granules but the delicate surface of the milled granules decrease their resistance to shear forces resulting in the reduced swelling ability and hence low peak viscosity values of the nano-starch granules. Set back and final viscosity was found lowest for the FSN indicating its lowest gelling ability and stability of the starch paste against retrogradation. Also the break down viscosity indicative of the disintegration degree of swollen starch granules was found lower in milled starch samples than native starch samples depicting their high resistance to shear thinning behaviour when cooked^38^. This is attributed to the prominent structural disintegration of the nano-starch samples resulting in the formation of linear chain molecules which pertains to reduced pasting parameters and gelation ability. Hoover et al.^39^ reports that the structural changes like crystallinity transformation, molecular order loss, amylopectin and amylose dispersion can significantly contribute to the reduction of viscosities and increase in the pasting temperature. Similar decrease in the pasting parameters were observed by Tan et al.^40^ for G80 corn starch samples after planetary ball milling at different times.

**Table 4:** Pasting properties and rheological parameters quantified by fitting the curve using Herschel–Bulkley equation and power law of native and nano starch samples.

Parameter	FS	FSN	SS	SSN
**Pasting properties**				
Peak viscosity	5617 ± 0.56^d^	2456 ± 0.78^c^	2282 ± 0.65^b^	1974 ± 0.98^a^
Trough viscosity	1052 ± 0.45^b^	993 ± 0.76^a^	1312 ± 0.67^c^	1484 ± 0.45^d^
Breakdown viscosity	4567 ± 0.56^d^	1463 ± 0.03^c^	970 ± 0.76^b^	490 ± 0.47^a^
Final viscosity	1335 ± 0.04^b^	1098 ± 0.05^a^	2804 ± 0.65^d^	2404 ± 0.57^c^
Setback viscosity	283 ± 0.55^b^	115 ± 0.32^a^	1497 ± 0.67^d^	920 ± 0.04^c^
Pasting temperature	79.2 ± 0.01^a^	85 ± 0.04^c^	84.8 ± 0.20^b^	87.85 ± 0.87^d^
**Hershel law**				
K	8.62	0.03	99.60	29.60
n	0.15	0.80	0.089	0.097
Ʈ_0_	2.546	0.026	53.20	− 12.74
R^2^	0.99	0.98	0.99	0.99
**Power law**				
K	5.95	0.03	42.13	14.6
n	0.20	0.79	0.19	0.26
R^2^	0.93	0.877	0.95	0.98

Ʈ_0_, K and n represents yield stress, consistency index, and flow behaviour index respectively. FS, FSN, SS and SSN represent native foxtail millet starch, nano foxtail millet starch, native sorghum starch and nano sorghum starch, respectively. Values expressed are mean ± standard deviation. Means in the row with different superscript (a, b, c & d) are significantly different at P ≥ 0.05.

### Rheological measurements

#### Mechanical spectra

**Figure 5:**
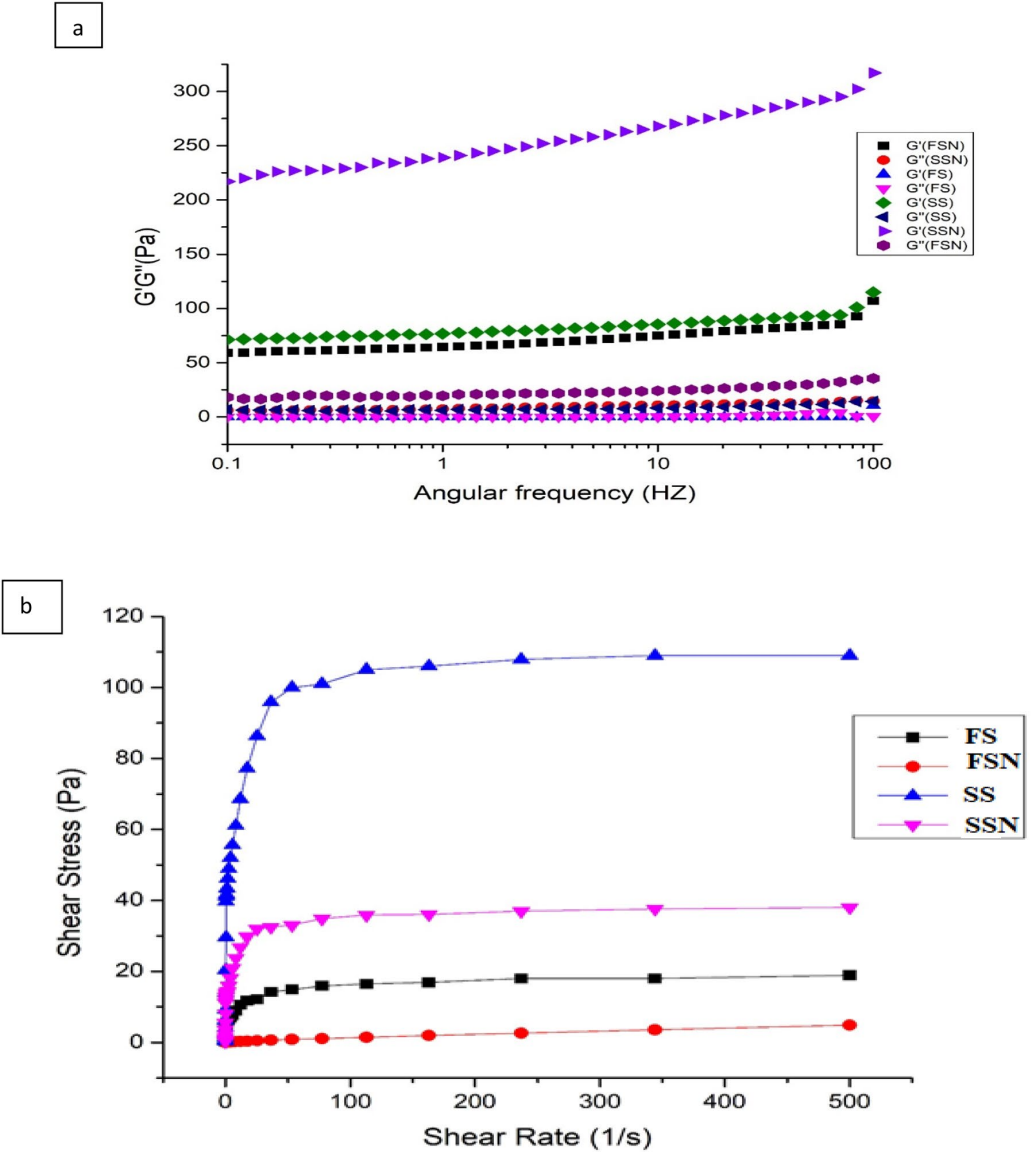
**(a,b)** Mechanical spectra measured as function of storage modulus (G´) and loss modulus (G´´) of starch samples **(a)** Flow curves of native and nano starch pastes **(b)**. FS, FSN, SS and SSN represents native foxtail millet starch, nano foxtail millet starch, native sorghum starch and nano sorghum starch, respectively.

The frequency sweeps were measured to determine the changes in storage modulus (G´) and loss modulus (G´´) over the frequency ranging from 0.1–100 Hz. The G´ (elastic modulus) was dominant over the G´´ (viscous modulus) in both native and nano-reduced starches throughout the frequency range which is indicative of its viscoelastic behaviour^41^ as shown in Fig. 5(a). The greater the difference between G´ and G´´ for a gel, higher is its elastic behaviour which was found for nano starch gels. The underlying reason might be the greater swelling ability of nano-reduced granules (as already discussed) during heating while the intact structure of native granules results in less water uptake and hence less visco-elastic response.

#### Steady shear properties

Plots of shear stress (s) versus shear rate data for native and nano-reduced starch pastes are shown in Fig. 5(b). The data for the determination of flow behaviour of millet starch pastes was fitted to two models viz. Herschel-Bulkley model and Power law for the evaluation of different rheological parameters like flow behaviour index (n), consistency index (K) and yield stress (Ʈ_0_) (Table 4). Co-efficient of determination (R^2^) value was found higher in Hershel bulkily model, thus can be considered best fitted for the evaluation of rheological parameters of millet starch gels. Overall the nano-reduced samples showed a proportional shear rate whereas native ones presented non-linear curves characteristic of pseudo-plastic flow behaviour fluid^42^. The yield stress values were found highest for native ones with maximum value for SS than FS. Similarly, FSN revealed higher yield stress than SSN. This indicates that the compact structure of native starches provides higher resistance to structure breaking at same shear rate. Nano-reduction resulted in a weak gel structure with higher ability to flow. The consistency index (k) indicative of strength of molecular association of the gels decreased in nano starch in the order of SS > FS > FSN > SSN indicating that native samples have more structural strength. Flow behaviour index (n) calculated from both the models were found to be less than one for all starch samples confirming shear thinning (Non-Newtonian) behaviour. Bhandari et al.^43^ attributed shear thinning behaviour to higher amount of breakage of the intra and inter molecular associative bonding system in starch network micelles due to shearing at high rates. The reduction in interchain association due to milling in nano-reduced samples might have resulted in lower gel network junction formation and less resistance to flow. Similar rheological behaviour was observed by Shah et al.^44^ in acetyl modified starch.

### Encapsulation efficiency

**Figure 6:**
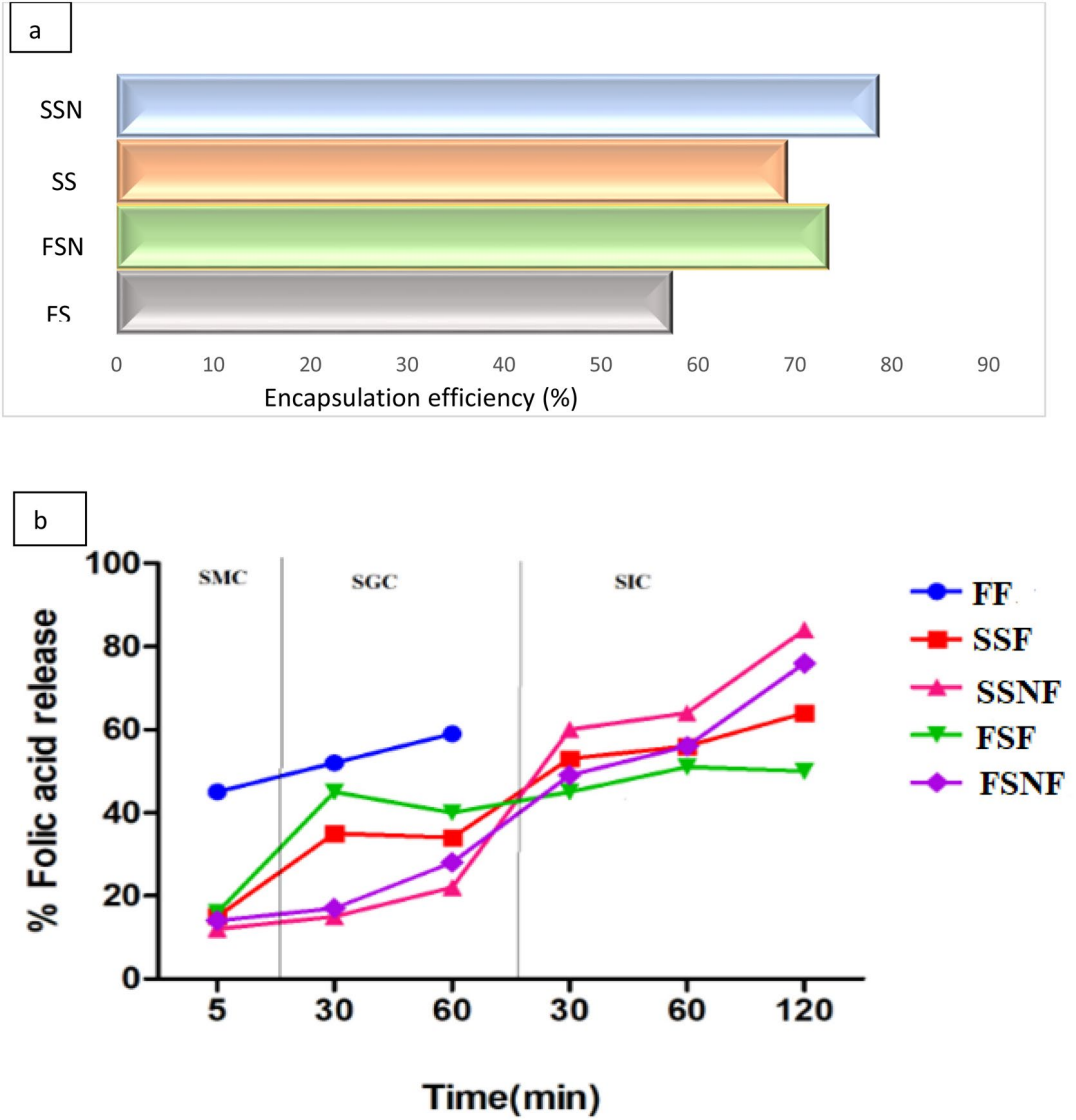
**(a,b)** Encapsulation efficiency **(a)** and in vitro release profile of folic acid **(b)** from native and nano-starch samples. FF, SS, SSNF, FSF and FSNF represent free folic acid, native sorghum folic acid, nano sorghum folic acid; native foxtail folic acid, nano foxtail folic acid, respectively. SMC, SGC and SIC represents simulated mouth conditions (5 min), simulated gastric conditions (60 min) & simulated intestinal conditions (120 min), respectively.

The encapsulating efficiency of native and nano reduced starch samples for the retention of folic acid is shown in the Fig. 6(a). The starch samples showed good encapsulating efficiency with SS revealed comparatively higher efficiency for folic acid encapsulation than FS. This might be due to the difference in the type of starch present, source of the starch, interaction between folic acid and starch. Ahmad et al.^45^ reported 50.20% folic acid encapsulation using horse chesnut starch matrix. The nano starch samples showed higher encapsulation efficiency than native starch samples. The efficiency for folic acid encapsulation increased to 78.76 and 73.43% for SSN and FSN. The encapsulating efficiencies are consistent with the particle size of the starch granules. Lower the size, better is the efficiency to form a film around the bioactive and retain the same for the further release at different target sites^46^.

### In-vitro release study

The nutraceutical activity of any bioactive is valid if it is released slowly and absorbed in the gastro-intestinal tract such that it gets absorbed in the blood stream and exhibit beneficial health benefits to the body. The in vitro release behaviour of folic acid encapsulated in native and nano-starch structures at different simulated conditions viz simulated mouth, gastric and intestinal conditions are depicted in Fig. 6(b). During the transit through simulated mouth conditions the folic acid release from encapsulated systems was found overall less when compared to free folic acid (45 µg/mL). The initial release from encapsulated structure might be attributed to the free bioactive present at the surface of the core material^47^. Under the simulated gastric conditions, the native starch samples showed significantly higher release than nano encapsulated systems. The SS sample showed a release of 34 µg/mL during the first 30 min while as FS released 45 µg/mL of folic acid. However, the nano-starch samples retained the bioactive and released only 15 µg/mL (SSN) and 17 µg/mL (FSN). During the next 60 min it showed a non-significant increase with FSN retaining the higher level of bio-active. This can be attributed to the fact that nano-reduced starch samples show high swelling index (as discussed earlier) thereby increasing the path length to diffuse through the nano-starch core material, thus protecting and retaining the bioactive under harsh conditions^48^. Similar gradual release pattern of curcumin was also observed from sago starch nanoparticles by Chin^49^. Another study also showed the similar results where beta glucan nanoparticles resisted gastric phase digestion more than native beta glucan for the target release of alpha tocopherol^50^. Under simulated gastric conditions the free folic acid was fully released with 52 µg/mL and 59 µg/mL released at 30 and 60 min. After this when the encapsulated systems are subjected to simulated intestinal conditions, with the increase in the time viz., 30, 60 and 120 min the bioactive carrier system degrades gradually and release of folic acid increased significantly. The nano starch carrier systems showed significantly high release than native carrier systems, with SSN displaying the highest release of 84 µg/mL after 120 min. The underlying reason might be that at higher pH and presence of bile salts significantly affected the nano-encapsulating systems due to their high surface area leading to rapid release of the folic acid. The results clearly reveal that nano-encapsulating systems are favourable for the delivery and target release of bioactives as compared to micro encapsulating system The similar increase in resveratrol release from zein/pectin core–shell nanoparticles under simulated gastric conditions was observed by Haung et al^51^.

## Materials and methods

Foxtail millet (*Setaria italica* L.) and Sorghum (*Sorghum bicolor* L.) were procured from local market of Srinagar, J&K, India. The samples were cleaned and foreign materials were removed manually and then stored at room temperature in air tight containers. The chemicals and reagents used for the study were of analytical grade and bought from Himedia Laboratories Pvt Ltd and Sigma Aldrich Pvt Ltd.

### Starch extraction

Starch was extracted according to the method described by Shah et al.^52^. Briefly, sorghum and foxtail millet seeds were pulverised in a domestic blender to obtain fine flour. Flour was then sieved through 50 µm mesh screen. The aqueous flour slurry (flour:water, 1:10) was then prepared and pH 9 was maintained. The slurry was then kept for 1 h and mixed at regular intervals. The mixture was then filtered through clean muslin cloth, followed by centrifugation at 3000×*g* for 15 min. The upper layer containing impurities was scrapped off while as lower starch was collected and washed three times with distilled water and then kept in hot air oven at 40 °C for drying.

### Nano-reduction of starch

Starch nano-reduction was carried out by the method of Jhan et al.^16^ with slight modifications. The starch was nano-reduced by ball milling method (Model: PM100, 117 Retsch, India). The starch (10 g) was kept in ceramic pot and ten 8 mm Zirconium balls were added and left for rotation at a speed of 600 rpm for 1 h.

### Zeta potential and particle size analysis of the starch

The particle size and zeta potential of starch samples was determined by following the method of Ahmad et al.^45^. Briefly, 0.01% w/v of sample was dispersed in mili-q water and sonicated in a bath sonicator for half an hour, particle measurement was then taken using a Zeta-sizer (Nano S, Malvern Instruments, Worcestershire, UK). For measuring the zeta potential, 0.01% of the sample was dispersed overnight in 0.1 Mm KCI at pH 6 in order to equilibrate the sample solution.

### Native and nano starch morphology

The basic morphological structure of starch samples was examined under vacuum conditions using Zeiss EVO 50 scanning electron microscope (SEM). The samples were analysed at 20 kV acceleration potential.

### X-ray diffraction (XRD)

The XRD patterns of the starch samples were recorded using a X’Pert PRO PANalytical, Netherlands, operated at 40 kV voltage and 35 mA current and X-ray line of λ = 1.5418 Å. The measurement range of diffractograms was recorded from 10 °C to 45 °C for 2θ at a step size of 0.02/min. The relative percent crystallinity (RC) of samples were evaluated using the equation of Rabek^53^.

$${\text{Relative crystallinity }}\left( \% \right)\, = \,{\text{A}}/{\text{A}}\, + \,{\text{C}}\, \times \,{1}00$$

where, A is the amorphous area and C is the crystalline area on the XRD curve.

### ATR-Fourier transforms infrared (FTIR) spectroscopy

The infrared spectra of starch samples were obtained using ATR FTIR spectrophotometer (CARY 630, Agilent Technologies, USA) within the range of 40–4000 cm^−1^ at a resolution of 4 cm^−1^.

### Gelatinization properties of starch samples

The gelatinization properties of starch samples was evaluated using a differential scanning calorimeter (DSC-1 STARe System, Mettler-Toledo) by the method of Gani^54^. Starch sample (3.5 mg, dry basis) was weighed in a hermetic platinum pan and diluted with distilled water (8 μL) with the help of micropipette. Prior to the measurement, the pan was allowed to stand for 1 h at room temperature. The pans were then heated at the temperature range of 20–250 °C at a rate of 10 °C/min. An empty platinum pan was used as a reference.

### Apparent amylose content

Apparent amylose content of the samples was evaluated by following the method of Ashwar^55^. 20 mg of starch sample was mixed thoroughly with 10 mL of KOH (0.5 M). The dispersion was then transferred to a volumetric flask (100 mL) and volume make up was done using distilled water. 10 mL of test starch solution was pipetted into another volumetric flask (50 mL) and 5 mL of hydrochloric acid (0.1 M) was added followed by addition of 0.5 mL of iodine reagent. The volume was finally diluted to 50 mL and the absorbance was measured at 625 nm (UV–visible Spectrophotometer, Model U2900 2JI-0003, Hitachi, Japan). The content of amylose present in the starch samples were determined using a standard curve which was developed using standard amylopectin and amylose blends from potato starch.

### Color

The color parameters of the starch samples was measured using Color Flex Spectrocolorimeter (Hunter Lab Colorimeter D-25, Hunter Associates Laboratory, Ruston, USA). Results were obtained in terms of L* (lightness), ranging from 0 (black) to 100 (white); a* (redness), ranging from + 60 (red) to − 60 (green) and b* (yellowness), ranging from + 60 (yellow) to − 60 (blue) values.

### Swelling power (SP) and solubility (S)

The swelling and solubility index for the starch samples was evaluated following the method of Shah et al.^32^ with slight modifications. Specifically, starch 0.25 g (W_0_) was dispersed in 10 mL of double distilled water and heated for 30 min at 60, 70, 80 and 90 °C . After this, the mixture was cooled at room temperature and centrifuged at 1500×*g* for half an hour. The supernatant was carefully removed and left over swollen starch sediment was weighed (W_1_) . The collected supernatant was dried at 105 °C in an oven until a constant weight (W_2_) was attained. Swelling power and solubility index of the starch samples were calculated using the equations given below.

$$\begin{gathered} {\text{Swelling power}} = \, \left( {{\text{W}}_{{1}} /{\text{W}}_{0} } \right) \, \times { 1}00 \hfill \\ {\text{Solubility index}} = \, \left( {{\text{W}}_{{2}} /{\text{W}}_{0} } \right) \times { 1}00 \hfill \\ \end{gathered}$$

### Emulsifying property

Emulsifying activity and stability was determined by the method described by Khan^41^. Starch suspension (1%) was mixed with 5 mL refined oil and homogenised for 15 min followed by centrifugation (5810, Eppendorf, Hamburg, Germany) at 1100*g* for 5 min The emulsion capacity and stability was calculated by the following equation

$${\text{Emulsifying capacity }}\left( \% \right){ = }\frac{{{\text{Height of the emulsi}}{\text{ed layer}}}}{{\text{Total height of tube contents}}} \times 100$$

$${\text{Emulsion stability}} \left( \% \right){ = }\frac{{{\text{Height of the emulsi}}{\text{ed layer after heating}}}}{{{\text{Height of the emulsi}}{\text{ed layer before heating}}}} \times 100$$

### Pasting property

A rapid viscos analyzer (RVA) (Tech Master, Perten Instruments Warriewood, Australia) was used to determine change in viscosity of starch samples during heating and cooling cycles as determined by Ashwar et al.^56^.

### Rheological measurement

The rheological properties of starch suspension was determined using rotational rheometer (MCR 102, Anton Paar) by the method of Shah et al.^44^. Briefly, 6 g of starch was dispersed in 100 mL distilled water and was heated at 90 °C for about 20 min with constant stirring. The gel formed was then left to cool for about 1 h. The flow properties like yield stress, flow behaviour index, and consistency coefficient was evaluated from the flow curve of shear rate (r) versus shear stress (t) by fitting the data into two rheological models i.e., Herschel-Bulkley model and Power law whose equations are given below.

1 $$\tau \, = \, \tau_{0} + {\text{ K}}\left( \sigma \right)^{{{\text{n}} }}$$

2 $$\tau = {\text{ K }}\left( \sigma \right)^{{\text{n}}}$$

where, τ (Pa) is the shear stress, τ_0_ (Pa) is the yield stress, K is the consistency index (Pa s^n^), and n is the flow behaviour index, σ is the shear rate.

To evaluate the visco-elastic range, amplitude sweep test was carried out for each starch gel with constant frequency of 1 Hz and shear stress ranging from 0.01 to 100 pa. The appropriate shear stress was selected i.e., 1 Pa, after linear viscoelastic range analysis. Frequency sweep test was then carried out at the frequency range of 0.1 to 100 Hz.

### Encapsulation

The encapsulation of folic acid in starch samples was carried out by following the method of Guevaraa et al.^12^ with slight modifications. Solution containing folic acid (10 mg) and acetone (50 mL) was prepared followed by the addition of starch samples (1 g) and kept for stirring at 600 rpm for 20 min. During this period, distilled water (100 mL) was added drop wise into the solution. The dispersion formed was kept on magnetic stirrer at room temperature until acetone gets fully evaporated. The resulting suspension was centrifuged at 4000 rpm for 40 min for the separation of nanoparticles with encapsulated folic acid. The obtained samples were washed three times with ethanol for the removal of excess folic acid. The resulting folic acid loaded starch samples were kept for drying in an oven at 30 °C for 2 days.

### Encapsulation efficiency

Encapsulation efficiency calculation was carried out by the method of Noor et al.^47^ using folic acid standard curve obtained with known concentrations having regression equation

$${\text{Y}} = \, 0.0{\text{667x }} + \, 0.00{33},{\text{ R}}^{{2}} = 0.{9955}$$

where x determines the content of folic acid present (µg/mL) and y determines the absorbance of the sample or standard solution of folic acid. Encapsulation efficiency of matrix i.e.; starch was calculated using following formulae:

$${\text{Encapsulation efficiency }}\left( \% \right) \, = \frac{{\text{Folic acid released from the matrix}}}{{\text{Total folic acid added initially}}} \times 100$$

### In-vitro sustainable release of particles

The folic acid release behaviour was studied under gastrointestinal conditions (GI) for macro amd nano starch samples as carrier systems. Folic acid loaded starch samples (10 mg) were suspended in simulated mouth conditions (10 mL). The conditions were prepared by dissolving a-amylase (0.2%) in phosphate buffered saline (PBS), pH 7.2. The sample and amylase solution was then vortexed for five minutes followed by centrifugation at 5000*g* for 10 min. The supernatant was collected and absorbance was measured using UV-spectrophotometer at 272 nm. Pellet was then recovered carefully and subjected to simulated gastric conditions by suspending the pellet in simulated gastric juice (10 mL). The conditions were prepared by dissolving 3 g /L pepsin (3 g/L) in sterile NacL solution (9 g/L) with adjusted pH to 3.0. After the intervals of 30 and 60 min, the solution was centrifuged at 10,000*g* for 5 min and absorbance was taken at 272 nm to measure the release of folic acid from macro and nano carrier systems. The pellet was again recovered and resuspended in simulated intestinal juice (10 mL), the conditions were prepared by dissolving bile salts (3 g/L) and pancreatin (10 g/L) in PBS. The samples were centrifuged (10,000*g* for 5 min) at 30, 60 and 120 min interval and absorbance was again taken at 272 nm. Percentage folic acid released was calculated by the formulae:

$$\% {\text{ Released folic acid }} = \frac{{{\text{Weight of folic acid}} {\text{ released}}}}{{\text{Weight of total encapsulated powder added}}} \times 100$$

### Statistical analysis

The experiments were carried out in triplicate and results were expressed in terms of mean ± standard deviation (n = 3). The data was analysed using commercial statistical package SPSS (IBM statistics 22) software and the significant difference was determined by analysis of variance (ANNOVA) at 95% confidence level.

## Conclusion

On nano-reduction, the starch granules showed enhanced functional attributes like high swelling and solubility index, better emulsion forming properties and low pasting parameters. The decreased retrogradation tendency and better thermal stability reflects its use in various food and non-food applications. Also, this physical technique effectively disrupted the supra molecular structure of starch granules creating nano structures with desired in-vitro release of folic acid in various gastric intestinal conditions which offer its potential application as nano-vehicle for target delivery and release of bioactives at the desired sites for the development of future functional foods and use in pharmaceutical industry. Thus, ball milling proved to be the powerful means for nano-sizing the starch granules. 
